# Faith as a Resource in Patients with Multiple Sclerosis Is Associated with a Positive Interpretation of Illness and Experience of Gratitude/Awe

**DOI:** 10.1155/2013/128575

**Published:** 2013-11-10

**Authors:** Arndt Büssing, Anne-Gritli Wirth, Knut Humbroich, Kathrin Gerbershagen, Sebastian Schimrigk, Michael Haupts, Klaus Baumann, Peter Heusser

**Affiliations:** ^1^Quality of Life, Spirituality and Coping, Institute of Integrative Medicine, Witten/Herdecke University, 58313 Herdecke, Germany; ^2^Freiburg Institute for Advanced Studies (FRIAS), Universität Freiburg, 79098 Freiburg, Germany; ^3^Department of Neurology, Communal Hospital Herdecke, 58313 Herdecke, Germany; ^4^Department of Neurology and Palliative Care, Köln-Merheim Hospital, 51109 Cologne, Germany; ^5^Neurological Hospital, Clinic of Lüdenscheid, 58515 Lüdenscheid, Germany; ^6^Augusta Hospital Anholt, Neurological Hospital, 46419 Isselburg, Germany; ^7^Caritas Science and Christian Social Work, Faculty of Theology, Albert-Ludwigs University, 79104 Freiburg, Germany; ^8^Institute of Integrative Medicine, Witten/Herdecke University, 58313 Herdecke, Germany

## Abstract

The aim of this cross-sectional anonymous survey with standardized questionnaires was to investigate which resources to cope were used by patients with multiple sclerosis (MS). We focussed on patients' conviction that their faith might be a strong hold in difficult times and on their engagement in different forms of spirituality. Consecutively 213 German patients (75% women; mean age 43 ± 11 years) were enrolled. Fifty-five percent regarded themselves as neither religious nor spiritual (R−S−), while 31% describe themselves as religious. For 29%, faith was a strong hold in difficult times. This resource was neither related to patients' EDSS scores, and life affections, fatigue, negative mood states, life satisfaction nor to Positive attitudes. Instead it was moderately associated with a Reappraisal strategy (i.e., and positive interpretation of illness) and experience of gratitude/awe. Compared to spiritual/religious patients, R−S− individuals had significantly (*P* < .0001) lower Reappraisal scores and lower engagement in specific forms of spiritual practices. The ability to reflect on what is essential in life, to appreciate and value life, and also the conviction that illness may have meaning and could be regarded as a chance for development was low in R−S− individuals which either may have no specific interest or are less willing to reflect these issues.

## 1. Introduction

There are several studies pointing to the fact that patients with chronic diseases may use their spirituality/religiosity (SpR) as a beneficial resource to cope [1–10], particularly patients with fatal diseases [11, 12]. However, less is known about relatively young patients with a chronic disease such as multiple sclerosis (MS) in this respect. Due to its often unpredictable course of exacerbations and remissions with significant impairment of both quality of life and life goals and due to the fact that there is no “cure” and only symptomatic and immunomodulatory therapy [13], patients often experience social isolation, are depressed, and thus have a higher risk of suicide [14–16]. 

A qualitative study among 7 patients with MS found that, during the course of disease, patients observed “positive changes in terms of their values and outlook” [17]. Interestingly, the patients reported that the disease associated with functional difficulties and psychological challenges was “ameliorated to some extent by an increased appreciation for life and spirituality” [17]. Also a further qualitative study enrolling 13 patients with MS found that adaptation to the disease was influenced by a variety of factors, including religion/spirituality [18].

First findings from cross-sectional studies indicate that spiritual/religious attitudes among patients with MS are significantly lower when compared to patients with cancer [3, 5], also their engagement in different forms of spiritual practices was significantly lower than in cancer patients [4]. Chen et al. [19] measured “overall belief and spirituality” in MS patients from the US and found that the spirituality scores were not related to age of diagnosis; moreover, most of the patients suggested a positive connection between spirituality and disability rather than a negative connection [19]. Makros and McCabe [20] found no significant association between spirituality/religiosity and psychological adjustment or quality of life among patients with MS; however, intrinsic religious orientation and quest religious orientation were associated with low psychological adjustment. To explain these surprising findings, Makros and McCabe [20] suggested that either patients utilized their religiosity (i.e., praying) to cope with their health affections and impaired quality of life, or they were more depressed because their religious activities did not result in the desired positive resolutions. Presumably, time plays a crucial role because even those patients with low interest in spirituality/religiosity might reactively “use” a spiritual source in acute situations (and quit when their expectations are not fulfilled), while during the long-lasting chronic course of illness there may occur religious developments and particularly those with a vital spirituality may continue to practice their religiosity whether this may have a beneficial effect on their health or not. 

Although the measures and patient samples are not comparable, it was obvious that in US patients with MS [19] the “overall belief and spirituality” score was relatively high (mean 4.1 ± 1.0, with a minimum of 1 and a maximum of 5), while German patients scored relatively low both on the (religious) Trust scale (mean 56.8 ± 28.0 in MS compared to the 70.0 ± 26.5 of the whole sample of chronic patients; maximum score 100) and also on the (spiritual) Search scale (mean 35.8 ± 2.5 compared to the 50.6 ± 25.9 of the whole sample; maximum scores 100) [3]. Thus, the relevance of spirituality to cope with MS may differ with respect to cultural and specific religious issues. In fact, Germany is a more secular society with about 42% of patients with chronic pain diseases who would regard themselves as neither religious nor spiritual [6], and this may have an impact on their strategies to cope with illness.

The aim of the study was thus to investigate (1) MS patients' conviction that their faith might be a “strong hold in difficult times”, and (2) the impact of patients' spiritual/religious attitudes on their life satisfaction, mood states, affections of daily life, internal adaptive coping strategies, and engagement in spiritual practices. We suggested that having faith is not related to the course of disease or life satisfaction but with the ways patients may view their life and how they cope with illness. 

## 2. Materials and Methods

### 2.1. Patients

We focussed on patients with multiple sclerosis (MS) because they are relatively young, and they have to deal with an illness which is characterized by an often unpredictable course of exacerbations and remissions with significant impairment of life goals and by the fact that there is no “cure.”

All individuals of this prospective, anonymous, multi-centre, cross-sectional study were informed about the purpose of the study, were assured of confidentiality and their right to withdraw at any time, and asked to provide informed consent. Ethical approval was obtained by the IRB of Witten/Herdecke University (number 21/2012). 

Outpatients with MS were consecutively recruited from four specialized hospitals, that is, Department of Neurology and Palliative Care, Köln-Merheim Hospital, Cologne; Department of Neurology, Communal Hospital Herdecke; Neurological Hospital Anholt, Clinic of Lüdenscheid; and Augustahospital Anholt, Isselburg. 

Inclusion criteria were verified diagnosis of multiple sclerosis, age between 18 and 65 years, and written consent to participate; exclusion criteria were manifest psychic diseases/affections (ICD10-classifications F0–F5).

### 2.2. Measures

#### 2.2.1. Adaptive Internal Coping Strategies

Adaptive coping strategies in response to MS were measured with the AKU questionnaire (AKU is an acronym of the German translation of “Adaptive Coping with Disease”), which was designed to identify adaptive coping styles, such as to create favorable conditions, search for information, medical support, religious support, social support, initiative spirit, and positive interpretation of disease [5, 21]. For this analysis we focused on internal resources rather than external resources, and used the following subscales.

- *Reappraisal: positive interpretation of illness* (Cronbach's alpha = .83) addresses a reappraisal attitude referring to cognitive processes of life reflection (i.e., reflect on what is essential in life; illness has meaning; illness as a chance for development; appreciation of life because of illness).
- *Conscious way of living* (alpha = .73) addresses cognitive and behavioral strategies in terms of internal powers and virtues (i.e., healthy diet; physical fitness; living consciously; keep away harmful influences; change life to get well).
- *Positive attitudes* (alpha = .68) refers to internal cognitive and behavioral strategies (i.e., realization of shelved dreams and wishes; resolving cumbering situations of the past; take life in own hands; doing all that what pleases; positive thinking; avoiding thinking of illness).

We added two single items addressing the attitudes towards their belief, that is, X1 “My faith is a strong hold in difficult times” and X2 “Because of my experiences I have lost my faith”.

All items were scored on a 5-point scale from disagreement to agreement (0: does not apply at all; 1: does not truly apply; 2: do not know (neither yes nor no); 3: applies quite a bit; 4: applies very much). The sum scores were referred to a 100% level (transformed scale score). Scores > 50% indicate high agreement or utilization of coping strategy, while scores < 50% indicate low usage of respective strategy. 

#### 2.2.2. Engagement in Spiritual Activities

To differentiate various forms of specific spiritual practices, we used the SpREUK-P questionnaire [4, 22]. The generic instrument was designed to measure the engagement in organized and private religious, spiritual, existential, and philosophical practices. In its shortened 17-item version it differentiates 5 factors [22]:

- religious practices (alpha = .82; i.e., praying, church attendance, and religious events, religious symbols);
- humanistic practices (alpha = .79; i.e., help others, consider their needs, do good, connectedness, etc.);
- existential istic practices (alpha = .77; i.e., meaning in life, self-realization, and get insight);
- gratitude/awe (alpha = .77; i.e., feeling of great gratitude, feelings of wondering awe, and experienced and valued beauty);
- spiritual (mind body) practices (alpha = .72; i.e., meditation, rituals, and working on a mind-body discipline (i.e., yoga, qigong, mindfulness, etc.)).

The items of the SpREUK-P are scored on a 4-point scale (0: never; 1: seldom; 2: often; 3: regularly). The scores can be referred to a 100% level (transformed scale score), which reflect the degree of an engagement in the distinct forms of a spiritual/religious practice (“engagement scores”). Scores > 50% indicate higher engagement, while scores < 50 indicate rare engagement.

#### 2.2.3. Spiritual/Religious Self Categorization

According to their responses to the SpREUK items f2.6 (“To my mind I am a religious individual” = R) and f1.1 (“To my mind I am a spiritual individual” = S), the practitioners were categorized as religious but not spiritual (R+S−), as not religious but spiritual (R−S+), as both religious and spiritual (R+S+), or as neither religious nor spiritual (R−S−) [3]. The respective items were scored on a 5-point scale from disagreement to agreement (0: does not apply at all; 1: does not truly apply; 2: do not know (neither yes nor no); 3: applies quite a bit; 4: applies very much). To avoid internal conflicts, we did not provide information how a religious or a spiritual individual should be defined. 

#### 2.2.4. Life Satisfaction

Life satisfaction was measured using the *Brief Multidimensional Life Satisfaction Scale* (BMLSS) [24] which uses items of Huebner's “Brief Multidimensional Students' Life Satisfaction Scale” [25, 26] and was tested among adults [24]. The eight items of the BMLSS address intrinsic (*Myself*, *Life in general*), social (*Friendships*, *Family life*), external (*School situation*, *Where I live*), and prospective dimensions (*Financial situation*, *Future prospects*). The internal consistency of the instrument was good (alpha = .87) [24]. For this analysis we used the 10-item version of the BMLSS which includes satisfaction with the health situation and satisfaction with the own abilities to manage daily life concerns. Moreover, we used three further items addressing satisfaction with the support by family, partner, or friends as an additional scale (“satisfaction with social support”). 

Each item was introduced by the phrase “I would describe my level of satisfaction as …,” and scored on a 7-point scale from dissatisfaction to satisfaction (0: terrible; 1: unhappy; 2: mostly dissatisfied; 3: mixed (about equally satisfied and dissatisfied); 4: mostly satisfied; 5: pleased; 6: delighted). The BMLSS sum score refers to a 100% level (“delighted”).

#### 2.2.5. Mood States

To assess mood states we relied on the 19-item ASTS (“Aktuelle Stimmungslage Skala”) scale of Dalbert [27] which refers to the profile of mood states (POMS) [28]. It measures the state component of subjective well-being and differentiates five mood states, that is, positive mood (6 items), sorrow (3 items), despair (3 items), tiredness (4 items), and anger (3 items). The internal consistency of the factors ranges from alpha = .83 to .94. The scale has a 7-point rating scale ranging from 0 (not at all) to 7 (very strong). 

#### 2.2.6. Multiple Sclerosis Associated Fatigue

To measure fatigue associated with MS, we used the “Fatigue Scale for Motor and Cognitive Functions” (FSMC) by Penner et al. [29]. This 20-item instrument has a very good internal consistency (alpha > .91). Ten items refer to the cognitive scale, and 10 items to the motoric scale which all were scored on a 5-point Likert scale ranging from 1 (does not at all) to 5 applies very much. FSMC sum scores ≥43 indicate mild fatigue, ≥53 moderate fatigue, and ≥63 strong fatigue. 

#### 2.2.7. EDSS Score

To classify the condition of the patients, we used the “Expanded Disability Status Scale” (EDSS). The EDSS is a method of quantifying disability in multiple sclerosis and monitoring changes in the level of disability over time [30]. The EDSS scale ranges from 0 to 10 in 0.5 unit increments that represent higher levels of disability. Scoring is based on an examination by a neurologist.

EDSS steps from 1.0 to 3.5 refer to people with MS who are able to walk unrestricted and are based on measures of impairment in eight functional systems, that is,

- pyramidal: weakness or difficulty moving limbs
- cerebellar: ataxia, loss of coordination, or tremor
- brainstem: problems with speech, swallowing, and nystagmus
- sensory: numbness or loss of sensations
- bowel and bladder function
- visual function
- cerebral (or mental) functions
- other.

Each functional system is scored on a scale from 0 (no disability) to 5 or 6 (more severe disability). 

EDSS steps from 4.0 to 9.5 are defined by the impairment to walking. 

#### 2.2.8. Self-Perceived Health Affections

Patients' self-perceived impairment of daily life though the health situation/disease (“health affections”) was measured with a visual analogue scale ranging from 0 (none) to 100 (unbearable). 

### 2.3. Statistical Analyses

Descriptive statistics as well as analyses of variance, first-order correlations, and regression analyses were computed with SPSS 20.0. We judged a *P* < .05 as significant; for correlation analyses we chose a significance level *P* < .001. With respect to classifying the strength of the observed correlations, we regarded *r* > .5 as a strong correlation, an *r* between .3 and .5 as a moderate correlation, an *r* between .2 and .3 as a weak correlation, and *r* < .2 as no or a negligible correlation.

## 3. Results

### 3.1. Characteristics of Enrolled Patients

Two hundred thirteen patients were enrolled in this study. 75% were women, and 22% were men (4% did not provide these data). Their mean age was 43 ± 11 years. Most were living with a partner (73%) and 27% were living alone (either single or divorced). 51% had relapsing remitting MS course, 25% progressive relapsing MS, and 23% chronic progressive MS. Their mean EDSS score was 3.7 ± 1.8, ranging from 0 to 7.5 (26% did not provide the respective data). All further socio demographic data are presented in Table 1. 

### 3.2. Attitudes towards Belief Ad Faith

The majority of patients had a Christian denomination (74%), 4% were Muslims, 3% had other denominations, and 18% none. With respect to their spiritual/religious self-categorization, 70% would not regard themselves as religious (Table 1); that is, 54% were neither religious nor spiritual (R−S−), 16% not religious but spiritual (R−S+), while 19% were religious but not spiritual (R+S−) and 12% both religious and spiritual (R+S+).

The statement “My faith is a strong hold in difficult times” was true for 29%, 52% rejected it, and 19% were undecided. Only 6% stated that they had lost their faith because of distinct experiences in life, 77% disagreed, and 17% were undecided. 

As shown in Table 2, the perception of “faith as a strong hold in difficult times” was not related to patients' health status, life satisfaction, negative mood states, or *positive attitudes*, while it correlated weakly with positive mood and *conscious way of living*; instead it was moderately associated with *reappraisal: positive interpretation of illness* and with patients' engagement in spiritual practices, particularly with *religious practices* and *gratitude/awe*. Having lost faith due to specific experiences in life was only weakly associated with reduced positive mood and low religious practices. In line with the aforementioned statement on faith as a resource, also patients' engagement in *religious practices* did not significantly correlate with health, mood, or life satisfaction, but moderately with *reappraisal: positive interpretation of illness* and *gratitude/awe* (Table 2).

With respect to the mean scores, those patients with faith as a strong hold have high scores on *reappraisal: positive interpretation of illness*, while those lacking this faith have *Reappraisal* scores which indicate a lack of a positive interpretation of illness (Table 3). In contrast, whether patients have this faith or not, they all have high scores for *conscious way of living*, *positive attitudes,* and life satisfaction. Engagement in spiritual/religious, existential, and humanistic practices was significantly higher in individuals with faith as a resource in difficult times and also with respect to *gratitude/awe* which is not an exclusive religious topic (Table 4).

Having this faith as a resource was not significantly influenced by gender, family status, educational level, or course of disease (data not shown). Instead, faith was a strong hold particularly for 67% of R+S+ and 60% of R+S− individuals, while of low relevance for R−S− (12%) and of minor relevance for R−S+ (6%) patients. 

### 3.3. Attitudes and Convictions of Nonreligious and Nonspiritual (R−S−) Patients

Patients with this R−S− attitude did not differ from their religious/spiritual counterparts with respect to their EDSS score, daily life affections, fatigue, life satisfaction, or positive mood (data not shown), while they had significantly lower *positive interpretation of illness* scores (Table 5). These R−S− patients had the lowest engagement in *religious practices*, *spiritual mind-body practices,* and *existential practices*, while the differences with respect to *humanistic practices* were significant only in trend (Table 6). Of interest was the fact that *gratitude/awe* was lowest in R−S− patient, and the highest in R+S+ patients. 

## 4. Discussion

Although it is not the “aim” of religion to generate well-being, several may nevertheless have the expectation that SpR is a resource to generate or at least maintain physical and mental health in cases of chronic illness. While it is true that SpR can be a resource to cope with chronic disease [1–12, 31], the current data indicate that even relatively young patients with MS regard their faith as a “strong hold in difficult” times. However, this attitude was not significantly related to the MS symptoms, course of diseases, daily life affections, fatigue, life satisfaction, or the development of *positive attitudes* as an adaptive coping strategy. Instead, patients with faith as a resource had significantly higher *reappraisal *strategies (i.e., *Interpretation of illness*), higher *gratitude/awe* scores, and somewhat better positive mood and *conscious way of living*. This means that their faith was not instrumentalized or reduced to a “tool” to restore health but was related to a more reflected look at what might be essential in life and to appreciate and value life despite the disease. Specifically, the scale *reappraisal: positive interpretation of illness *addresses patients' ability to reflect on what is essential in life; that illness may have meaning; that illness may be regarded as a chance for development; and to an appreciation of life because (or despite) of illness. Similarly, the scale *gratitude/awe* deals with the frequency patients experience strong feelings of gratitude, feelings of wondering awe, and how often they experienced and valued beauty in life. Both scales are moderately intercorrelated (*r* = .42) and implicitly address patients' ability to face life as it is. It was striking that these scales were moderately related to faith and to a religious attitude; in fact, engagement in *religious practices* showed the same correlation pattern as the statement on faith as a strong hold in difficult times. 

What might be of relevance for patients which may have access to such a resource is not necessarily true for those lacking faith as a resource or who regard themselves as neither religious nor spiritual (R−S−). While these a-religious/sceptic patients did not differ with respect to daily life affections, fatigue, life satisfaction, or positive mood, they have lower abilities for a *positive interpretation of illness*, which was significantly higher in R+S+ and R−S+ individuals. Also *gratitude/awe* was significantly lower in R−S− patients with MS; moreover, they were less engaged in *existential practices*. This again indicates that interest in or openness for spiritual/religious issues may have an influence on how patients cope with illness and how they perceive and value their life and open their mind for others. This ability was low in R−S− individuals: they may have either no specific interest or are less willing to reflect these issues. How these individuals could be supported requires further exploration.

An important argument could be that particularly R−S− patients might suffer from cognitive impairments which thus could result in lower abilities to reflect and value life. Tinnefeld et al. [32] found that cognitive deficits may occur in patients with MS even in the absence of physical affections. Also Schulz et al. [33] pointed to the fact that even in the early stages of MS one may find discrete cognitive impairments. However, the patients enrolled in this study had moderate disability scores (mean EDSS scores 3.7 ± 1.8; 33% with EDSS scores 4.0 to 6.5, and 2% with scores > 6.5), and among them neither the EDSS scores nor self-perceived daily life affections, fatigue, life satisfaction, or positive mood did significantly differed between R−S− and SpR patients. 

A limitation of this study was the cross-sectional design, which does not allow for causal interpretations; longitudinal studies are needed to substantiate the findings of this study. Moreover, a further limitation is that we recruited outpatients with rather moderate EDSS scores. Most of them have a normal daily life and thus may “ignore” their underlying disease. With respect to the categorized EDSS scores (see Table 1), patients with higher EDSS scores had higher fatigue scores (*F* = 9.3; *P* < .0001) and daily life affections (*F* = 17.0; *P* < .0001), and were more tired (*F* = 2.9; *P* = .037), while the other psychometric variables did not significantly differ (data not shown). 

Further analyses with high-maintenance patients with progressive courses of disease are needed. 

## 5. Conclusion

Although spirituality/religiosity is a relevant strategy to cope also in relatively young individuals with MS, faith as a resource was not significantly associated with mood states, course of disease, or life satisfaction. Instead, this resource was associated with their ability to reflect on what is essential in life, with the conviction that illness may have meaning and could be regarded as a chance for development, and to appreciate and value life. A recent systematic review found that there is evidence that specific approaches of mind-body medicine (i.e., yoga, mindfulness, relaxation, and biofeedback) might be helpful to ameliorate MS symptoms [34]. Particularly yoga and mindfulness training improved MS fatigue with low side effects. Both approaches can be regarded as secular forms of spirituality (although they can be found in specific religious contexts, too) which might be of interest for the majority of a-religious patients with MS because these interventions focus awareness on the self, environment, interaction with others, and life style. In fact, at least in healthy individuals within a 6-month yoga practice, a significant increase of specific aspects of spirituality (i.e., conscious interactions/compassion, religious orientation) and mindfulness can be observed [23]. Particularly R−S− individuals showed moderate effects for an increase of such conscious interactions (with others, self, and nature) and compassion. In contrast, religious individuals may find hope and hold in their faith, and related engagement in individual forms of religiosity (i.e., private prayers, meditation, rituals) and/or organized forms of religiosity (i.e., church attendance). Further research in this direction is needed. 

## Figures and Tables

**Table 1:** Characteristics of 213 patients with MS.

Variables*	Mean/%
Gender, %	
Women	78
Men	22
Age, years (mean, SD)	42.6 ± 11.4
Family status, %	
Married	56
Living with partner	17
Divorced	8
Single	19
Educational level, %	
Secondary (Hauptschule)	22
Junior high school (Realschule)	33
High school (Gymnasium)	36
Other	9
Religious orientation, %	
Christian	74
Other	8
None	18
Spiritual/religious self-perception, %	
Neither religious nor spiritual (R−S−)	54
Not religious but spiritual (R−S+)	16
Religious but not spiritual (R+S−)	19
Both religious and spiritual (R+S+)	20
Employment status, %	
At pension	32
Unable to work	7
Unemployed	3
House work	8
Self-employed	4
Employed (business)	46
Course of MS, %	
Relapsing remitting	51
Progressive relapsing	25
Chronic progressive	23
EDSS score (mean, SD; range)	3.7 ± 1.8 (0–7.5)
0.0–1.5 (%)	10
2.0–3.5 (%)	29
4.0–6.5 (%)	33
7.0–10 (%)	2
No information (%)	26
Self-perceived health affection (mean ± SD, range)	42.3 ± 21.5 (0–90)

*Data refer to the responding patients (i.e., 8 individuals did not state their gender, or 20 individuals did not provide information on their course of MS).

**Table 2:** Correlation analyses.

	Faith is a strong hold in difficult times	Religious practices (SpREUK-P)
Faith is a strong hold in difficult times		.705**
Have lost my faith		−.216**
Health status		
EDSS score	.084	−.052
Perceived daily life affections (VAS)	.020	.067
Cognitive fatigue (FSMC)	.013	.101
Motoric fatigue (FSMC)	.071	.118
Fatigue sum score (FSMC)	.042	.115
Mood status (ASTS)		
Sorrow	−.049	.144
Despair	−.071	.069
Tiredness	.080	.153
Positive mood	.185**	.092
Life satisfaction (BMLSS-10)		
Life satisfaction sum score	.121	−.016
Satisfaction with social support	.028	.016
Adaptive (internal) coping strategies (AKU)		
Conscious way of Living	.192**	.148
Positive attitudes	.087	.029
Reappraisal: positive interpretation of illness	.408**	.306**
Engagement in spiritual practices (SpREUK-P)		
Existential practices	.202**	.256**
Humanistic practices	.183**	.237**
Religious practices	.705**	
Spiritual mind-body practices	.272**	.282**
Gratitude/awe	.477**	.498**

**P < .01 (Pearson).

**Table 3:** Having faith and associations with health status, life satisfaction, and adaptive coping strategies.

Faith is a strong hold in difficult times	Conscious way of living (AKU)	Positive attitudes (AKU)	Reappraisal: positive interpretation of illness (AKU)	Life satisfaction (BMLSS-10)	Positive mood (ASTS)	EDSS scores
No	69.8 ± 18.6	70.5 ± 16.8	38.2 ± 23.2	68.6 ± 18.3	23.3 ± 7.7	3.5 ± 1.7
Undecided	72.3 ± 13.4	68.0 ± 17.0	50.1 ± 23.9	66.5 ± 15.0	24.3 ± 7.0	4.2 ± 1.8
Yes	77.3 ± 17.4	74.7 ± 14.4	60.9 ± 26.5	72.8 ± 16.1	26.5 ± 6.9	3.8 ± 1.8

All patients	72.4 ± 17.4	71.0 ± 16.4	47.1 ± 26.0	69.3 ± 17.1	24.4 ± 7.4	3.8 ± 1.8

*F* value	**3.5**	2.3	**16.5**	1.9	**3.3**	1.6
*P* value	**.031**	n.s.	**<.0001**	n.s.	**.040**	n.s.

Results are means ± standard deviation. No: does not apply at all/does not truly apply; undecided: do not know (neither yes nor no); yes: applies quite a bit/applies very much.

**Table 4:** Having faith and engagement in spiritual practices/activities.

Faith is a strong hold in difficult times	Existential practices (SpREUK-P)	Humanistic practices (SpREUK-P)	Religious practices (SpREUK-P)	Spiritual mind-body practices (SpREUK-P)	Gratitude/awe (SpREUK-P)
No	40.0 ± 26.2	62.5 ± 20.3	7.2 ± 10.1	14.0 ± 19.1	35.5 ± 19.3
Undecided	47.8 ± 21.1	63.0 ± 18.5	30.2 ± 15.5	27.5 ± 26.2	44.8 ± 15.4
Yes	53.1 ± 29.0	71.1 ± 14.3	45.1 ± 27.7	28.3 ± 28.0	60.0 ± 22.8

All patients	45.4 ± 26.5	64.9 ± 18.8	22.6 ± 24.0	20.9 ± 24.3	44.0 ± 21.9

*F* value	**4.9**	**4.3**	**87.5**	**8.9**	**27.3**
*P* value	**.009**	**.015**	**<.0001**	**<.0001**	**<.0001**

Results are means ± standard deviation. No: does not apply at all/does not truly apply; undecided: do not know (neither yes nor no); yes: applies quite a bit/applies very much.

**Table 5:** Spiritual/religious self-categorization and associations with health status, life satisfaction and adaptive coping strategies.

	Conscious way of living (AKU)	Positive attitudes (AKU)	Reappraisal: positive interpretation of illness (AKU)	Life satisfaction (BMLSS-10)	Positive mood (ASTS)	EDSS scores
R+S+	76.4 ± 13.3	72.3 ± 13.3	71.6 ± 18.5	66.9 ± 18.6	24.8 ± 6.4	3.4 ± 1.8
R+S−	71.5 ± 17.5	70.1 ± 14.4	47.4 ± 26.3	74.1 ± 13.1	26.8 ± 7.3	3.3 ± 1.8
R−S+	78.7 ± 20.0	80.3 ± 15.2	60.8 ± 23.0	70.1 ± 16.4	23.9 ± 7.7	3.6 ± 1.7
R−S−	71.1 ± 17.1	69.6 ± 17.9	40.1 ± 22.7	67.3 ± 17.8	24.1 ± 7.8	3.8 ± 1.7

All patients	73.0 ± 17.4	71.7 ± 16.7	48.4 ± 25.4	69.0 ± 17.0	24.7 ± 7.5	3.6 ± 1.8

*F* value	1.8	**3.4**	**14.2**	1.5	1.2	0.8
*P* value	n.s.	**.020**	**<.0001**	n.s.	n.s.	n.s.

Results are means ± standard deviation.

**Table 6:** Spiritual/religious self-categorization and engagement in spiritual practices/activities.

	Existential practices (SpREUK-P)	Humanistic practices (SpREUK-P)	Religious practices (SpREUK-P)	Spiritual mind-body practices (SpREUK-P)	Gratitude/awe (SpREUK-P)
R+S+	63.4 ± 23.8	71.1 ± 13.6	53.0 ± 27.4	36.7 ± 21.7	60.8 ± 21.8
R+S−	43.9 ± 23.3	66.4 ± 16.9	39.1 ± 19.4	15.6 ± 21.1	54.2 ± 19.8
R−S+	69.8 ± 22.0	69.9 ± 18.7	15.8 ± 23.1	44.0 ± 28.6	53.1 ± 24.1
R−S−	37.3 ± 21.8	61.8 ± 19.1	12.7 ± 14.1	14.1 ± 18.9	36.1 ± 18.4

All patients	46.7 ± 25.7	65.0 ± 18.3	22.8 ± 23.7	21.8 ± 24.4	45.2 ± 22.2

*F* value	**19.7**	2.5	**37.6**	**18.5**	**14.3**
*P* value	**<.0001**	.060	**<.0001**	**<.0001**	**<.0001**

Results are means ± standard deviation.
